# Extraction and characterization of a pectin from sea buckthorn peel

**DOI:** 10.3389/fnut.2022.969465

**Published:** 2022-09-05

**Authors:** Yulian Zhu, Keshan Liu, Michael Yuen, Tina Yuen, Hywel Yuen, Qiang Peng

**Affiliations:** ^1^College of Food Science and Engineering, Northwest A&F University, Yanling, China; ^2^Puredia Limited, Xining, China; ^3^Beijing Engineering and Technology Research Center of Food Additives, Beijing Technology and Business University, Beijing, China

**Keywords:** sea buckthorn peel, pectin, gelation property, characterization, emulsifying property

## Abstract

Sea buckthorn peel is the by-product of the sea buckthorn processing, which contains many bioactive compounds. In this paper, sea buckthorn high methoxyl pectin (SBHMP) was obtained, with a yield of 8% and a light-colored. The SBHMP was a high methoxyl with a degree of esterification of 57.75% and uronic acid content of 65.35%. The structural and morphological characterization of SBHMP were analyzed by high-performance liquid chromatography, Fourier-transform infrared spectroscopy, and scanning electron microscopy. Results showed that SBHMP presented a sheet and layered stacked morphological, and was mainly composed of galacturonic acid, arabinose, galactose, rhamnose, and mannose, which indicated that SBHMP mainly consisted of homogalacturonan (HG) and rhamnogalacturonan-I (RG-I) type pectin polysaccharides. In addition, SBHMP also presented significant gel, thickening, and emulsifying properties. The results exhibited that SBHMP could form jelly-like gels under acid and high sucrose conditions, presenting a shear-thinning behavior and increasing apparent viscosity with the enhancement of pectin and sucrose contents. Besides, SBHMP could form oil-in-water emulsions with pectin concentrations of 1.0–3.0%. When the SBHMP concentrations were 2.0 and 3.0%, the emulsions were stable during 7 days of storage. Findings in this paper demonstrated the potential of SBHMP to be a food thickener and emulsifier and support the in-depth utilization of sea buckthorn by-products.

## Introduction

As a natural biomolecule, pectin is an essential regulator of biological modifiers and is widely used in biochemistry, food, and pharmaceutical industries (1, 2). Due to their high safety, bioactivities, and biodegradability, natural pectin has recently been extensively studied (3). Numerous studies have shown that plant pectin has various biological activities such as antioxidant (4), antitumor (5), and regulation of intestinal microorganisms (6). Pectin is also widely used as a functional food additive due to its unique emulsification and gelation properties (7). According to its DE values, pectin is classified as high methoxyl pectin (HMP DE>50%) and low methoxyl pectin (LMP DE<50%). Compared to LMP, HMP can form a gel network with high sugar and acid environment, and the gelation is insensitive to Ca^2+^. The existence of H^+^ inhibits the dissociation of carboxyl, and the abundance of sugar help to reduce the hydration radius of pectin, resulting in the interaction force between molecules weaker and chains closer. Besides, the hydrophobicity of methyl can also promote the interaction between chains (3). The unique gel-forming mechanism and solution environment allow HMP to be used as a food thickener in a wider range of applications.

Sea buckthorn (*Hippophae rhamnoides*) belongs to the Elaeagnaceae family, and is widely cultivated in China, India, Mongolia, and Europe because of its drought, cold, and salt resistance (8). As a medicinal and edible homologous plant, sea buckthorn has many related pharmacological effects recorded in writings as early as the Tang and Ming dynasties (9). In recent years, relevant studies have shown that extracts of sea buckthorn berries, leaves, and seeds contained a lot of biological activities (10, 11). With the development of the sea buckthorn industry, pomace, peel, seed meal, and other processing by-products have also accumulated, but they have not been fully utilized, resulting in wasted resources.

In recent years, the extraction of pectin from plant by-products has attracted interest due to its low toxicity and biological activity. According to relevant studies, a large amount of pectin is contained in the peel of fruits and vegetables, and the composition and application characteristics of pectin from different sources are quite different (12, 13). Currently, the study on sea buckthorn pectin was limited, and the application characterization of sea buckthorn pectin was unclear. In addition, the bioactive components in sea buckthorn vary in different growth habitats, among which the content and function of bioactive components in sea buckthorn grown in plateau tend to be better than those in plain (14). Therefore, the study of plateau sea buckthorn peel as an important source of pectin and the characterization of its functional properties are of great significance for the development of natural food additives.

This study aims to extract pectin from sea buckthorn peel, effectively improve its color and make it more in line with the market requirements. The gel properties, rheological properties, and emulsifying properties were also studied to develop Sea buckthorn peel pectin as a new natural food additive.

## Materials and methods

### Materials

Sea buckthorn peel was obtained from Puredia Limited (Xining, Qinghai, China). The sea buckthorn peel was the by-product of the production of sea buckthorn juice. The sea buckthorn fruits were squeezed to get the juice, then the residue was washed and dried, and the sea buckthorn peel was sorted out. The standard monosaccharides were bought from Solaribio Life Sciences Co., Ltd., (Beijing, China). The uronic acid (D-galacturonic acid) was obtained from Aladdin Biochemical Technology Co., Ltd., (Shanghai, China). The ethylene diamine tetraacetic acid (EDTA), citric acid, and sodium ascorbate were all edible grade and purchased from Man Pong Industry Company Limited (Henan, China). All the other chemicals and solvents used in this study were analytical grade.

### Preparation of sea buckthorn pectin

According to the previous method (15), sea buckthorn peel was mixed with distilled water at a 1:10 *m/V* liquid-material ratio, 0.5‰ citric acid, 0.5‰ sodium ascorbate, and 0.2‰ EDTA. The pH of the mixture was then adjusted to 2.0 with HCl (1 mol/L) and incubated at 80°C for 1 h. After the incubation, the mixture was collected by centrifugated at 6,500 rpm for 15 min to collect the supernatant and further enrich it with filtration. Then the supernatant was concentrated by rotary evaporation and added 0.0004‰ volume of sodium metabisulfite to decolorize it slightly. Subsequently, the solution was mixed with 1.5 volumes of ethanol and stored for 4 h. SBHMP was obtained after dialyzed and lyophilization.

### Chemical characterization

#### Determination of uronic acid content

According to the previous method (16), the uronic acid content of SBHMP was determined using the carbazole-sulfuric acid method with galacturonic acid as a standard. And the absorbance of the solution was measured at 528 nm with a spectrophotometer (model UV7, METTLER TOLEDO, Zürich, Switzerland).

#### Degree of esterification

The degree of esterification (SBHMP) of SBHMP was determined by the method of Ma et al. (17). The DE was calculated using the following Equation:


$$\mathrm{DE}\left(\%\right)=\frac{{\text{V}}_{2}}{{\text{V}}_{1}+{\text{V}}_{2}}\times 100$$

Where:

V_1_ and V_2_ demoted the volume of NaOH consumed for the first and second titrations.

#### Determination of monosaccharide composition

Referring to the method by Mzoughi et al. (18), the monosaccharide composition of SBHMP was determined by pre-column 1-phenyl-3-methyl-5-pyrazolone-derived high-performance liquid chromatography (PMP-HPLC) with Agilent 1100 system (Zorbax Eclipse Plus-C18 column, 4.6 mm×250 mm, 5 μm, Agilent, United States). The mobile phases were phosphate buffer (pH 6.6) and acetonitrile with a flow rate of 1.0 mL/min, and the column temperature was 30°C.

### Structural characterization

#### Fourier-transform infrared spectroscopy analysis

SBHMP and KBr were mixed at the ratio of 1:100 to prepare the disk and detected at the wavelength from 400 to 4,000 cm^–1^ with an FTIR instrument (Vetex70, Bruker Co., Ettlingen, Germany).

#### Scanning electron microscopy

The surface morphology of SBHMP was obtained from an S-3400N scanning electron microscope (Hitachi, Tokyo, Japan). The freeze-dried SBHMP was attached to a sample stage and plated with gold by a sputter coater. Images of SBHMP were collected at 100×, 500×, and 2,000×.

### Thermal analysis

According to the previous by Lin et al. (19) with slight modifications, the thermal property of SBHMP was detected by a differential scanning calorimetry (Q2000, Waters, Milford, MA, United States). The SBHMP sample (3 g) was loaded into an aluminum crucible and sealed immediately. Meanwhile, an empty aluminum crucible was used as a reference. The detection was carried out under the N_2_ environment at the flow rate of 50 mL/min, the temperature was raised at the speed of 30°C/min, and the scanning range of temperature was 30–300°/min.

### Gel properties

#### Gel preparation

The gel of SBHMP and sucrose was prepared according to a previous method (20). SBHMP was dissolved with distilled water at 70°C and adjusted the pH to 2.5, the solutions with different concentrations of sucrose (40–60% *m/m*) were prepared and stored at room temperature overnight. In addition, another group of solutions with different pH (2.0–3.5) was also prepared.

#### Line spread test

Referring to the previous study of Ke et al. (21) with some modifications, the gelation of SBHMP was evaluated by line spread teat on an acrylic plate. The plated was covered with several concentric circles with a spacing of 0.4 cm, and the diameter of the middle circle was 2.8 cm. Subsequently, the sample was positioned in the center of the plate and allowed to flow at room temperature for 2 min. The spread values of each sample were recorded rapidly after flow.

#### Frequency scanning of sea buckthorn high methoxyl pectin gel

According to the previous study by Rafe and Razavi (22), the rheological characterization of SBHMP gel was carried on a rheometer (DHR-1, Waters, United States) with a 40 mm plate and a 0.1 mm gap. The gel was prepared according to the results of the line spread test and performed as follows: (1) A strain scan (0.02–20%) at an angular frequency of 1 rad/s was first performed to determine the linear viscoelastic zone. (2) A frequency sweep of 0.1–100 rad/s was performed in the linear viscoelastic region (1%) and recorded the storage modulus (G′) and loss modulus (G′′). The G′ and G′′ of frequency sweep were calculated as the following Equations:


$${\text{G}}^{'}\;=\;{\text{K}}^{'}\;\times \;\omega^{\text{a}}$$

$${\text{G}}^{"}\;=\;{\text{K}}^{"}\;\times \;\omega^{\text{b}}$$

Where k′ (k′′) and a (b) refer to the constant and frequency constant, and ω is the angular velocity.

### Rheological measurements

The effect of SBHMP concentration (1.0–4.0% m/V), sucrose concentration (10–40% m/V), CaCl_2_ (0.05–0.3% m/V), and pH (3.0–7.0) on the rheological characteristic of pectin solution were measured according to a relative study (23). All solutions were prepared and stored at room temperature overnight. The rheological properties were measured by a rheometer (DHR-1, Waters, United States) in a 40 mm parallel plate with a gap of 1 mm. The analyses were carried out at 25°C for 120 s with a shear rate from 0.01 to 100 s^–1^.

### Emulsifying properties

#### Preparation of emulsion

The SBHMP emulsion was prepared according to the previous method with some modifications (24). Different concentrations (1.0, 1.5, 2.0, and 3.0% m/V) of SBHMP were prepared and mixed with corn germ oil at an equal volume. The mixtures were fully homogenized by a high-speed homogenizer (T18, IKA, Germany) at 10,000 rpm for 2 min to obtain the emulsion. Another group of emulsions with different concentrations (10, 20, 30, and 40% m/V) of corn germ oil and 2.0% SBHMP were also prepared to investigate the effect of oil fraction on the emulsifying properties.

#### Evaluation of emulsion properties

The emulsions were prepared and stored at room temperature for 7 days. The morphology of emulsions was observed using a fluorescent inverted microscope (LX71, Olympus, Japan) on the first day. Besides, the heights of the mixture and emulsion phase were recorded during the storage, and the creaming index (CI) was calculated as the following Equation (25):


$$\text{CI}=\frac{H_{t}}{H_{e}}\times 100\%$$

Where the Ht was the height of the total mixture and the He was the height of the emulsion phase.

#### Particle size evaluation

The particle size of the emulsion was determined using a laser particle size analyzer (LS3320, Beckman Coulter, United States) on the first and last day. The particle size distribution and average particle size of the emulsion were recorded during the measurement.

### Statistical analysis

A one-way fixed-effects analysis of variance (ANOVA) test was performed using statistical software (SPSS 18.0, SPSS Inc., Chicago, IL, United States). All trials in this study were done in triplicate, and the statistical means and standard deviations were calculated and shown.

## Results and discussion

### Preparation of sea buckthorn pectin polysaccharide

**FIGURE 1:**
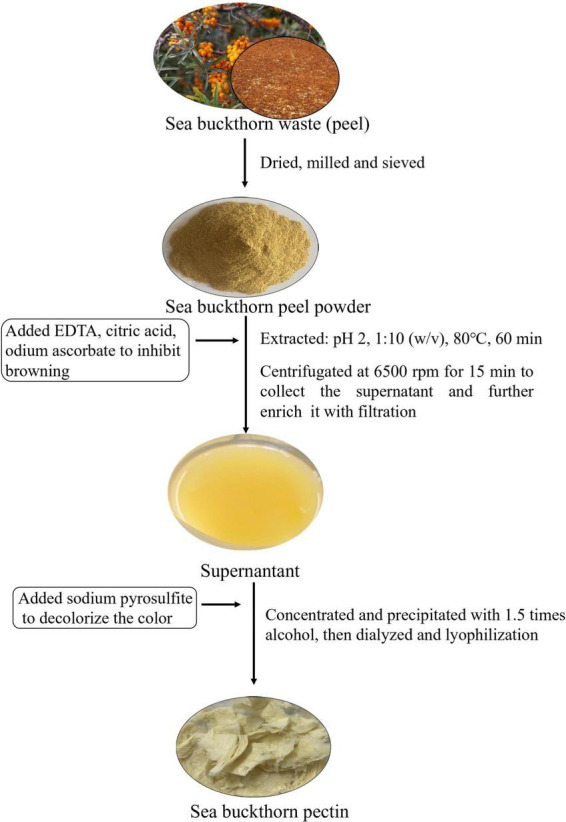
The flow chart of the preparation of sea buckthorn pectin.

The flow chart of sea buckthorn pectin polysaccharide (SBHMP) was shown in Figure 1. Pectin is a group of water-soluble polysaccharides, which exists in the network formed by cellulose and hemicellulose in the plant cell wall (26). Heat solution treatment could disrupt the interaction between plant cell wall, dissolve the pectin polysaccharide into solution, and then add ethanol or metal salt to precipitate it (27). In this study, SBHMP was also prepared with acid and alcohol. Sea buckthorn peel is the by-product of the process of sea buckthorn juice, and therefore, impurities like metal ions may be mixed in during the process, making SBHMP a deepening color (28). Besides, acidic and heat conditions during the extraction of SBHMP may promote the phenomena of Maillard and Caramelization, thus influencing the product’s color. Metal chelators could effectively bind metal ions associated with the browning of polysaccharides during extraction (29, 30). The addition of sodium ascorbate in the preparation process can prevent the oxidation of the extract. Relevant studies have shown that combing a small number of multiple browning inhibitors can effectively reduce the color of plant material (31–33). Therefore, citric acid, sodium ascorbate, and EDTA were added as browning inhibitions in the extraction of SBHMP. The final yield of SBHMP was 8%, with a light color and great market acceptability.

### Chemical characterization of sea buckthorn pectin

#### Degree of esterification

The degree of esterification (DE) is one of the most important characteristics of pectin and is closely related to its functional properties such as solubility and gelation. The size and type of DE are influenced by the source and preparation of pectin (34). The DE of SBHMP was 57.75%, indicating that SBHMP was high methoxyl pectin (HMP), indicating that SBHMP could form a stable gel under high sugar and acidic environment (35).

#### Content of uronic acid

This study determined the uronic acid content of SBHMP by the carbazole-sulfuric acid method. This method dehydrated the uronic acid produced by the hydrolysis of SBHMP in H_2_SO_4_ to form a furfural derivative, which could be condensed with carbazole to form a purplish red substance with maximum absorbance at 528 nm. Besides, there was a linear relationship between the absorbance of the reaction system and uronic acid content (16). As shown in Table 1, the uronic acid content of SBHMP was 65.35%, indicating that SBHMP was a typical pectin polysaccharide, while the specific monosaccharide components required further study.

**TABLE 1:** Chemical composition of SBHMP.

Physicochemical features	SBHMP
Uronic acid (%)	65.35
DE (%)	57.75
**Monosaccharides (%)**	
Mannose (Man)	3.18
Rhamnose (Rha)	4.50
Glucuronic acid (Glc A)	0.64
Galacturonic acid (Gal A)	66.49
Glucose (Glc)	2.31
Galactose (Gal)	5.73
Xylose (Xyl)	0.73
Arabinose (Ara)	15.35
Fucose (Fuc)	1.07

### Structural analysis

#### Monosaccharide composition

**FIGURE 2:**
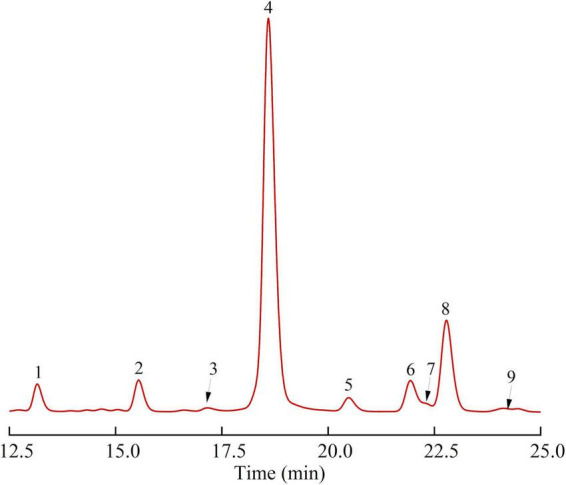
The monosaccharide composition of SBHMP (1 Mannose, 2 Rhamnose, 3 Glucuronic acid, 4 Galacturonic acid, 5 Glucose, 6 Galactose, 7 Xylose, 8 Arabinose, and 9 Fucose).

As shown in Table 1 and Figure 2, SBHMP was mainly composed of 9 monosaccharides, galacturonic acid, arabinose, galactose, rhamnose, mannose, glucose, fucose, xylose, and glucuronic acid, and the molar ratio was 66.49: 15.35: 5.73: 4.50: 3.18: 2.31: 1.07: 0.73: 0.64. From the results of monosaccharide composition, SBHMP may be classified as a complex consisting of homogalacturonan (HG) and arabinogalactan (RG-I) type pectin polysaccharides. The high content of galacturonic acid indicted the existence of homogalacturonan, which is mainly composed of GalA in the main chain and classified as an acid polysaccharide (3). Besides, the richness of arabinose and galactose suggested that SBHMP contained them in the branched chains of RG-I and free arabinogalactan as the neutral polysaccharide (36).

#### Fourier-transform infrared spectroscopy analysis

**FIGURE 3:**
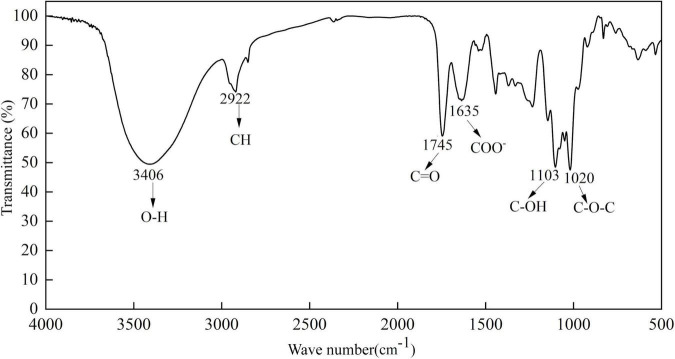
FTIR spectra of SBHMP.

FTIR is one of the common methods used for the structural characterization of carbohydrates, the FTIR spectrum of SBHMP is shown in Figure 3. The results showed that SBHMP was a typical macromolecular compound linked by hydroxyl-rich monosaccharides through glycosidic bonds (37), thus, there was a strong and broad absorption peak in 2,406 cm^–1^. Due to the large number of -CH_2_ in the sugar ring, the obvious peak at 2,922 cm^–1^ was the stretching vibration of -CH (27). The peak intensity around 1,745 cm^–1^ was related to the vibration of the asymmetric tension of carbonyl methyl ester. The peak near 1,635 cm^–1^ belonged to the vibration of the symmetric tension of carboxylate ions. Besides, the peak area at 1,745 and 1,635 cm^–1^ could reflect the DE of pectin. According to Figure 3, the peak at 1,745 cm^–1^ was stronger than 1,635 cm^–1^, indicating that SBHMP was high in methoxy pectin (4), the same as the results of the DE measurement. The peaks at 1,103 and 1,020 cm^–1^ were probably due to the stretching vibrations of C-OH in the branched chains and C-O-C in the glycosidic bonds (15). Other peaks from 500 to 1,000 cm^–1^ were the characteristic adsorption of the pyran rings in SBHMP (27).

#### Scanning electron micrograph analysis

**FIGURE 4:**
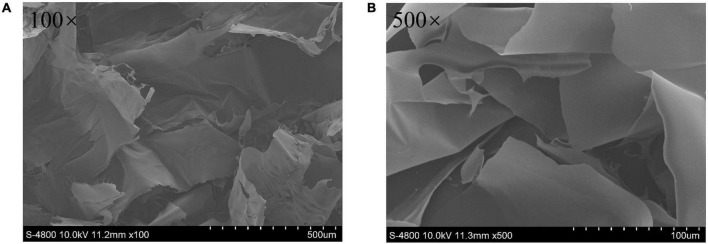
SEM images of SBHMP with the magnification of **(A)** 100×, and **(B)** 500×.

The morphological property of SBHMP was detected by SEM, and the results were shown in Figure 4. It could be concluded that SBHMP presented a sheet and layered stacked structure with a smooth and dense surface. Such a particular structure provided SBHMP with a larger surface and higher solubility for SBHMP. In addition, the lamellar structure of SBHMP made it easier to combine with other substances, which might provide some specific properties (15). These findings were familiar with the pectin polysaccharide extracted from jujube pomace, which also exhibited a smooth surface and a lamellar shape (19).

### Thermal analysis results

**FIGURE 5:**
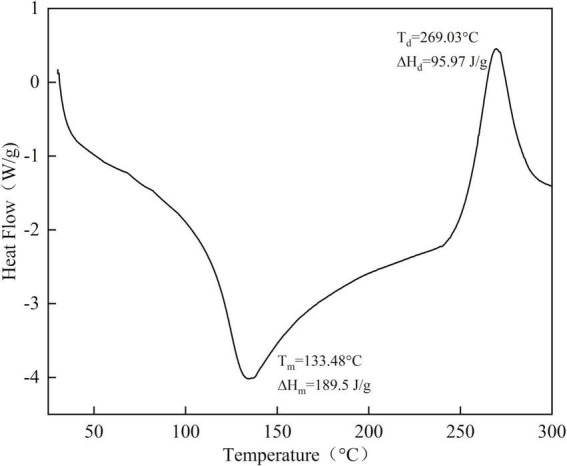
DSC thermogram images of SBHMP.

To further study the thermodynamic properties of SBHMP, its thermal stability was evaluated by differential scanning calorimetry, and the result was shown in Figure 5. The DSC diagram has two main peaks: the heat absorption peak and the heat release peak. The heat absorption peak is the melting temperature and the area of the peak is the enthalpy change of melting. The exothermic peak is the degradation temperature, and the area of the peak is the enthalpy change of degradation (38). The melting temperature of SBHMP was 133.48°C, corresponding to the enthalpy of melting of 189.5 J/g, and the enthalpy of degradation was 269.03°C, corresponding to the enthalpy of degradation of 95.97 J/g. The results indicated that SBHMP started to melt at 133.48°C and decompose at 269°C. As the temperature increased, the water molecules within the sample began to disperse, and oxidation and aggregation reactions occurred. With further heating, the galacturonic acid chains within the pectin started to break and degrade, the glycosidic bonds were broken, the carboxylic acid groups underwent decarboxylation reactions, and the structure was drastically altered (39). The higher enthalpy change of melting and stability of SBHMP may be related to the higher degree of esterification and uronic acid content. The higher heat absorption peak indicates that SBHMP had superior water retention and a higher content of hydrophilic groups (19). This was consistent with the results shown by SEM, where the larger surface area and lamellar stacked structure of SBHMP may allow for higher adsorption property.

### Gel property analysis

#### Line spread test

**FIGURE 6:**
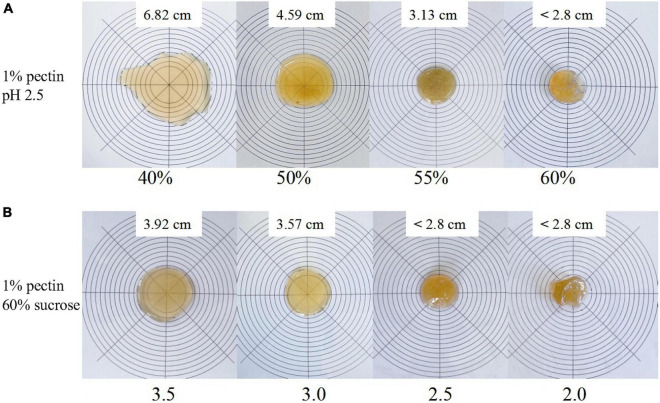
The visual aspect of SBHMP gel in line spread tests with various concentrations of sucrose, and under different pH values: **(A)** effects of various concentrations of sucrose on gel morphology **(B)** effect of different pH values on gel morphology.

Gelation is one of the most important properties of pectin, and the gelation mechanism of SBHMP as an HMP is closely related to its structure and formation conditions. The high sugar content and low pH facilitate the reduction of intermolecular distances between pectin, and the formation of hydrogen bonds, making pectin molecules form a network structure. The hydrated sucrose molecules can also adsorb in the gaps of the network structure through hydrogen bonding and intermolecular forces, forming a stable gel structure (40). Thus, in this study, the effects of different sucrose contents and pH values on the gelation of SBHMP were studied. As shown in Figure 6A, with the increase in sucrose content, the fluidity of the mixture decreased. Under the condition of 1% SBHMP, 55% sucrose, and pH 2.5, a honey-like gel could be developed (with a diameter of 3.1 cm). When the sucrose content increased to 60%, a jelly gel with a certain elasticity could be formed (with a diameter less than 2.8 cm). While with the decrease of sucrose content below 50%, the fluidity of the mixture increased, and gel could not be formed (with a diameter of more than 4.0 cm). And when the sucrose content was reduced to 40%, the fluidity further enhanced the viscosity of the mixture system and promoted the formation of a stable network structure. The gel formation of HMP is also related to the pH in the mixture. Pectin generally has a negative charge in the solution, and when the pH is below 3.5, the charge in the system can be neutralized. Then with the addition of a binding substance, the highly hydrated pectin dehydrated to form a network structure (41). Therefore, the different gelation states formed at a fixed 1% SBHMP, 60% sucrose, and a pH of 2.0–3.5 were investigated in this study. The results in Figure 6B showed that the gelation of SBHMP improved as the pH decreased, with reduced fluidity and increased stability. When the pH is at 2.5 and 2.0 stable gels could be formed (with a diameter less than 2.8 cm). Due to the decrease in pH, the electrostatic interactions between pectin molecular chains and the groups inner the chains increased, resulting in an improvement in the gels’ hardness, viscosity, and stability (42). The conclusion could be obtained that a stable gel of SBHMP could be formed at the condition of 1% SBHMP, 60% sucrose, and the pH at 2.0 or 2.5. While the specific rheological properties of the gels need further study.

#### Rheological property

**FIGURE 7:**
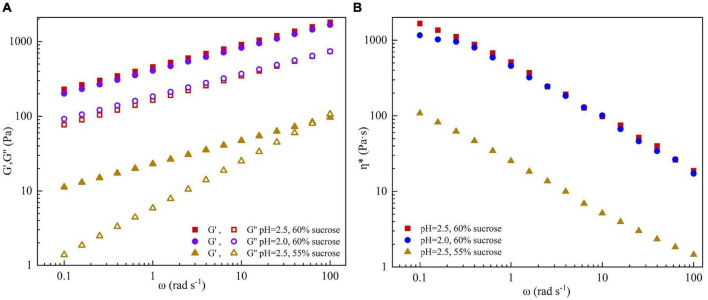
The effect of different concentrations of sucrose and pH values on **(A)** storage modulus (G′), loss modulus (G′′), and **(B)** complex viscosity (η*, c) of SBHMP gels.

Based on the results of the line spread test, three groups of SBHMP gels with great gelation were prepared as follows: fixed the content of SBHMP at 1%, added 60% sucrose at pH 2.5 (group A), added 60% sucrose at pH 2.0 (group B) and added 55% sucrose at pH 2.5 (group C), respectively. The rheological properties of A, B, and C can be summarized according to Figure 7. The storage modulus (G′) is related to the energy lost in elastic deformation, which reflects the elasticity of the gel. The loss modulus (G′′) is related to the energy lost in irreversible deformation, which reflects the viscosity of the gel. The relative magnitudes of G′ and G′′ represent the viscoelastic characteristics of the sample (43). As shown in Figure 7A, G′ values were always higher than G′′ in groups A and B, indicating a solid-state. Group C′s G′ and G′′ values were lower than groups A and B. With the increase of ω, the curves of G′ and G′′ dominated, and the sample changed from a biased solid-state to liquid, with a significant difference between a and b values and poor frequency stability. The unstable gelation might be due to the decreased sucrose content of group C, which increased the water activity of the mixture, reduced the viscosity, and enhanced the fluidity (41, 44). From Figure 7B, the complex viscosity η* of three groups decreased with increasing share rate, indicating that they were typical non-Newtonian fluids that exhibited share thinning properties. Compared to group A, B exhibited smaller viscosity values at low shear rates, which was consistent with the state of the gel in Figure 7B. However, as the shear rate increased, the viscosity change tended gradually to the same, indicating that they exhibited similar rheological properties at a high shear rate. It could infer that changes in pH have no significant effect on the gel properties of SBHMP in processing, which may facilitate its usage in the food industry. The viscosity decreased significantly with decreasing sucrose concentration in group C compared to group A, which was familiar with the results in Figure 5. This indicated that the effects of sucrose content change on the mixture’s viscosity were more obvious than that of pH change within the range of conditions in this study.

### Rheological measurement of sea buckthorn high methoxyl pectin

In recent years, attention has been paid to the research on the rheological properties of food colloids, which are closely related to their functional properties. Compared to other colloidal solutions, the flow characteristics of pectin solutions at low concentrations approximate Newtonian fluid, while exhibiting the properties of the pseudoplastic fluid at high concentrations (21). The viscosity of pectin solutions is not only related to its structural properties but is also influenced by the concentration of pectin in solution, pH, and ion concentration (43).

#### Influence of sea buckthorn high methoxyl pectin concentration on rheological properties

**FIGURE 8:**
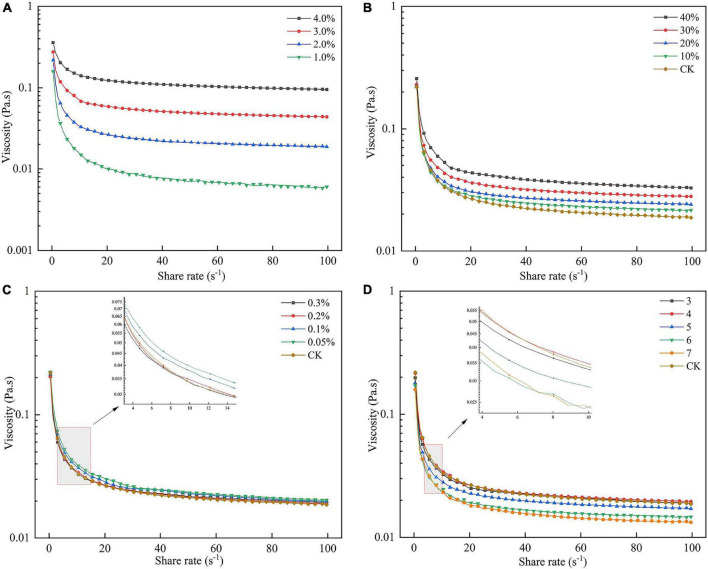
Effect of **(A)** various SBHMP concentrations, **(B)** various sucrose concentrations, **(C)** various CaCl_2_ concentrations, and **(D)** different pH values on the pectin rheological properties.

As shown in Figure 8A, with increasing shear rate, the apparent viscosity of all solutions decreased and showed shear thinning behavior, indicating that the SBHMP solution was a typical non-Newtonian fluid. Besides, as the pectin concentrations increased, the viscosity of solutions increased, and the shear thinning resistance behavior was enhanced. The apparent viscosity of 1, 2, 3, and 4% SBHMP solutions were 0.07, 0.11, 0.17, and 0.27 Pa at the shear rate of 1 s^–1^, respectively. This might be due to the increase in SBHMP concentration, the number of pectin molecules increased, and the intermolecular force increased, resulting in more viscoelastic chain structures. The viscosity of SBHMP was higher than that of gabiroba pectin (with the apparent viscosity of 0.02 and 0.13 Pa at concentrations of 1 and 3%) and sweet lemon peel pectin (with the apparent viscosity below 0.01 and 0.1 Pa at the concentrations of 1 and 2%) at the same shear rate (45, 46). This showed that SBHMP had better rheological properties, which expanded its potential as a stabilizer and thickener in the food industry.

#### Influence of sucrose concentration on rheological properties

Figure 8B exhibited that the apparent viscosity of SBHMP solution was enhanced with the increasing sucrose concentration. This might be caused by the sucrose-containing many hydrophilic groups, which would compete with SBHMP for water molecules in solution, reducing the activity of water molecules and leading to a decrease in the solubilization of pectin molecular chains and an enhancement of interaction (46). In this study, when the sucrose concentration reached 40%, it could significantly enhance the shear-thinning resistance of SBHMP solution, indicating that SBHMP could play a better thickening role in high sugar foods.

#### Influence of CaCl_2_ concentration on rheological properties

It could be seen from Figure 8C that the addition of low concentrations of Ca^2+^ (0.05 and 0.1%) could increase the apparent viscosity of the SBHMP solution. This may be because Ca^2+^ formed calcium bridges between carboxyl groups of pectin molecules to enhance the network structure and the aggregation state, thus increasing the apparent viscosity. However, due to the higher DE of SBHMP, the effect of Ca^2+^ concentration on its viscosity was limited. When the Ca^2+^ concentration further increased to 0.2% and above, the viscosity of the SBHMP solution did not change significantly. This might be related to the high Ca^2+^ concentration reducing the repulsive force between molecules in solution, resulting in the agglomeration of pectin molecules (47).

#### Influence of pH on rheological properties

It could be summarized from Figure 8D, the apparent viscosity of SBHMP solutions varied with the increasing pH. The viscosity was highest in the pH range at 3–4, and slightly decreased at the same shear rate at pH 5. While the apparent viscosity decreased significantly when the pH reached 6 and 7, and the shear thinning behavior increased. It indicated that SBHMP was more stable when the pH was below 5 and was suitable as a thickener for low-acid products. This might be because the strongly acid condition allowed the carboxyl groups in the SBHMP structure to dissociate into carboxylate ions and the intermolecular repulsion (48). With the increase of pH, the carboxyl group dissociation in the SBHMP molecules was inhibited in the weakly acidic environment, the intermolecular hydrogen bonding force was enhanced, and the solution viscosity was larger. When the pH increased further, the number of H^+^ decreased and the number of -OH increased in the solution. The -OH groups interacted with pectin molecules, causing the SBHMP structure to curl and intermolecular forces to weaken, ultimately leading to a decrease in solution viscosity (49).

### Emulsifying properties

Carbohydrates such as pectin can form a hydration layer in aqueous oil solutions to prevent oil droplets from collecting. The greater the thickness of the hydration layer formed, the better the emulsion stability (50). The emulsification capacity and emulsion stability of pectin make it a valuable tool for the food industry. The emulsification properties of pectin are closely related to its hydrophobic group content, structural characteristics, and molecular weight (51).

#### Creaming index evaluation

**FIGURE 9:**
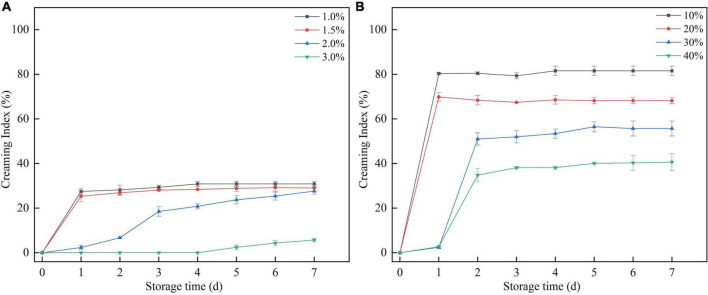
The creaming index of the mixtures during storage: **(A)** effect of various SBHMP concentrations on the CI values, **(B)** effect of various sucrose concentrations on the CI values.

The mixture of SBHMP and corn oil was stirred at high speed to form a stable and homogeneous emulsion, with no breakage or phase separation within a short time after preparation. After 7 days of storage, phase separation occurred in varying degrees in the individual blends. When the oil phase volume was fixed, the emulsion separation decreased as the concentration of SBHMP increased from 1.0 to 3.0%. Emulsification diminished when the SBHMP concentration was fixed as the oil phase concentration increased (10–40%). The creaming index of each sample during storage can be seen in Figure 9. The emulsions in the 3.0% group did not emulsify during the storage period of days 1–4. The emulsion index increased slowly from day 4–7 days to 5.69% on day 7, which was lower than that of the other concentrations. This indicated that the emulsification property of pectin is closely related to its concentration. As the concentration of SBHMP increased, the interfacial tension decreased, promoting the formation of emulsified particles. Moreover, the cross-linking of the pectin chain structure of SBHMP in the mixed system is enhanced, forming a greater spatial site resistance, thus preventing the aggregation of oil droplets (52). While, when the SBHMP concentration was fixed, the emulsion index increased significantly after 1 day of storage when the corn oil content increased from 10 to 20%, indicating that rapid and obvious phase separation occurred. The emulsion index increased significantly after 2 days of storage when the corn oil content was 30 and 40%, and the phase separation was completed.

#### Observation of droplet

**FIGURE 10:**
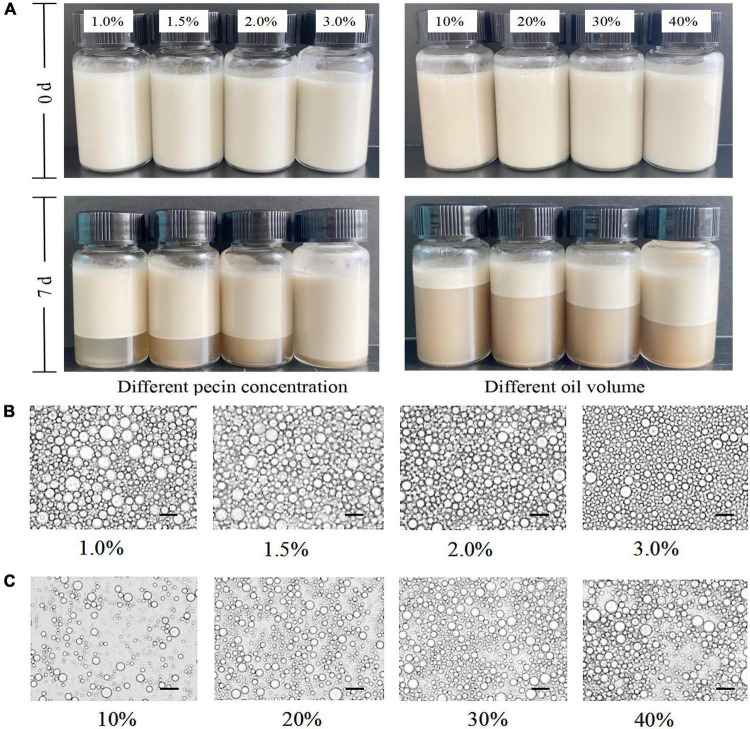
**(A)** Digital photos of oil-in-water emulsions stabilized by different concentrations of pectin and different volumes of oil. Optical micrographs of the emulsions stabilized by various contents of **(B)** SBHMP, and **(C)** oil.

The state of the emulsion droplets formed in different conditions could be observed in Figure 10. It could be seen that the emulsion system was an oil-in-water, in which the diameter of the emulsion droplets decreased with the increase of SBHMP concentrations and the distribution was more uniform, indicating that SBHMP played a good emulsification role in the mixed system. It was probably due to the increase of SBHMP concentrations, the viscosity of the emulsion system increased, and the stability of the emulsion enhanced. While as the oil content increased, the oil droplet aggregated, the emulsion particles were larger, and the emulsion stability decreased (53).

#### Droplet size distribution

**FIGURE 11:**
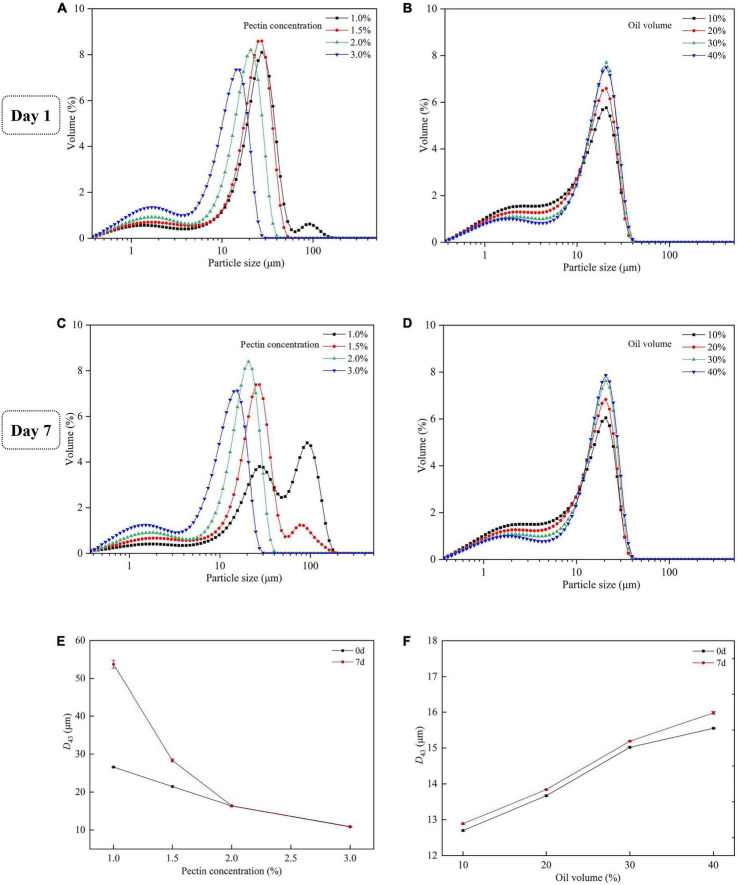
Droplet size distributions of emulsions on the first day **(A)** with various concentrations of SBHMP, and **(B)** with different volumes of oil. Droplet size distributions of emulsions after 7 days of storage **(C)** with various concentrations of SBHMP, and **(D)** with different volumes of oil. The *D*_43_ values of the emulsions during the storage **(E)** with various concentrations of SBHMP, and **(F)** with different volumes of oil.

The droplet size distribution and the *D*_43_ of emulsion with different concentrations of SBHMP and oil were shown in Figure 11. As the SBHMP concentrations increased, the main peak of the droplet size distribution plot shifted to the left, with *D*_43_ gradually decreasing from 26.57 μm (with 0.1% SBHMP) to 10.82 μm (with 0.3% SBHMP), indicating the formation of more small droplets, which is consistent with the microscopic observations. At the concentration of SBHMP of 1.0%, a small number of droplets over 100 μm were produced, whereas at 2.0% of SBHMP, the droplets were all less than 40 μm, indicating that SBHMP played a stronger emulsifying role in the emulsion system as the concentration increased. After 7 days of storage, the particle size distribution of the group with the addition of 1.0 and 1.5% SBHMP changed significantly, with new peaks appearing and a significant increase in *D*_43_. The position of the main peak in the addition of 1.0% SBHMP also changed, indicating that emulsion breakage might have occurred in the mixture. The changes in droplet size distribution again indicated that the emulsification of SBHMP was concentration-dependent. In the emulsification, pectin not only covered the oil droplets but also influenced the emulsification process through its viscosity effect (54). Therefore, the potential of SBHMP as a good rheology modifier needs to be further investigated. Although the change in oil phrase volume did not effectively affect the position of the main peak of the particle size distribution, the area of the peak increased slightly with increasing oil phase volume, with the droplets particle size (*D*_43_) increased from 12.70 μm (with 10% oil) to 15.55 μm (with 40% oil). This indicated that the size of the droplet was positively correlated with the oil phase content. As the oil phase content increased, the water-oil interfacial tension decreased, droplet aggregation occurred, and the homogeneous stability of the emulsion decreased (15). It also suggested that the spatial barrier formed by SBHMP at different oil phase contents can inhibit emulsion breakage and maintain emulsion stability in a short period.

## Conclusion

In summary, SBHMP was extracted and decolorized from sea buckthorn peel at a yield of 8%. The structural analysis exhibited that SBHMP was a high methoxyl pectin with a smooth and layered morphology. SBHMP was a typical pectin with a DE of 57.75% and a high uronic acid content of 65.35%. Besides, SBHMP was mainly composed of galacturonic acid, arabinose, galactose, rhamnose, and mannose, which could be classified as a complex consisting of HG and RG-I type pectin polysaccharides. The gel properties evaluation exhibited that SBHMP could form a jelly-like gel with an acid and high sucrose condition, exhibiting a shear thinning behavior. The apparent viscosity changed according to the pH values, pectin content, and sucrose concentration. The emulsifying properties measurement showed that SBHMP could form oil-in-water emulsions with different concentrations of SBHMP, and when SBHMP concentration reached 2.0% the emulsions were stable during the storage with the *D*_43_ values not changing more than 0.5 μm. This study supported that SBHMP could be a natural food additive, and further research should be devoted to exploring its specific functions.

## Data availability statement

The original contributions presented in this study are included in the article/supplementary material, further inquiries can be directed to the corresponding author.

## Author contributions

YZ: formal analysis, investigation, data curation, and writing— original draft preparation. KL: methodology, validation, visualization and funding acquisition, and writing—review and editing. QP: conceptualization, funding acquisition, and project administration. All authors contributed to the article and approved the submitted version.
